# Environmental factors influence both abundance and genetic diversity in a widespread bird species

**DOI:** 10.1002/ece3.856

**Published:** 2013-10-28

**Authors:** Yang Liu, Simone Webber, Katharine Bowgen, Lucie Schmaltz, Katharine Bradley, Peter Halvarsson, Mohanad Abdelgadir, Michael Griesser

**Affiliations:** 1 State Key Laboratory of Biocontrol and School of Life Sciences, Sun Yat-sen UniversityGuangzhou, 510275, China; 2 Evolutionary Ecology Group and Computational and Molecular Population Genetics, Institute of Evolution and Ecology, University BernBalzerstrasse 6, Bern, CH-3012, Switzerland; 3 Centre for Ornithology, University of BirminghamEdgbaston, Birmingham, BT15 2TT, UK; 4 School of Applied Sciences, Bournemouth University, Talbot CampusPoole, Dorset, BH12 5BB, UK; 5 Animal Ecology Group, Centre for Ecological and Evolutionary Studies, University of GroningenPO Box 11103, Groningen, 9700 CC, The Netherlands; 6 10 Springhill Close, London, SE5 8AJ, UK; 7 Section of Animal Ecology, Department of Ecology and Evolution, Evolutionary Biology Centre, Uppsala UniversityUppsala, SE-75236, Sweden; 8 Department of Biology, College of Sciences, University of HailHail, PO 2440, Saudi Arabia; 9 Department of Ecology, Swedish University of Agricultural SciencesUppsala, Sweden; 10 Anthropological Institute and Museum, University ZürichZürich, 8057, Switzerland

**Keywords:** Animals, conservation, molecular ecology, population genetics

## Abstract

Genetic diversity is one of the key evolutionary variables that correlate with population size, being of critical importance for population viability and the persistence of species. Genetic diversity can also have important ecological consequences within populations, and in turn, ecological factors may drive patterns of genetic diversity. However, the relationship between the genetic diversity of a population and how this interacts with ecological processes has so far only been investigated in a few studies. Here, we investigate the link between ecological factors, local population size, and allelic diversity, using a field study of a common bird species, the house sparrow (*Passer domesticus*). We studied sparrows outside the breeding season in a confined small valley dominated by dispersed farms and small-scale agriculture in southern France. Population surveys at 36 locations revealed that sparrows were more abundant in locations with high food availability. We then captured and genotyped 891 house sparrows at 10 microsatellite loci from a subset of these locations (*N* = 12). Population genetic analyses revealed weak genetic structure, where each locality represented a distinct substructure within the study area. We found that food availability was the main factor among others tested to influence the genetic structure between locations. These results suggest that ecological factors can have strong impacts on both population size per se and intrapopulation genetic variation even at a small scale. On a more general level, our data indicate that a patchy environment and low dispersal rate can result in fine-scale patterns of genetic diversity. Given the importance of genetic diversity for population viability, combining ecological and genetic data can help to identify factors limiting population size and determine the conservation potential of populations.

## Introduction

Understanding factors that drive population size is central to ecology, population genetics, and conservation biology (Backwell et al. 1998; Frankham et al. 2002; Taft et al. 2002). Given the continuing impact that anthropogenic activities are having on habitats and ecosystems, many species are suffering from declining population sizes (Beerens et al. 2011). It is therefore crucial for conservation management to have insight into the ecological factors that drive population size, if we are to mitigate for the negative effects of human activity. A key reason why population size is central to conservation biology is that it correlates with genetic diversity, which serves as a basis of the evolutionary potential of a species (Frankham et al. 2002; Reed and Frankham 2003). A number of evolutionary processes such as selection, gene flow, and historical demography affect the genetic diversity in a population (Hayes and Fox 1991; Boettcher et al. 1995; Bazin et al. 2006). The genetic diversity of individuals within a population affects a range of ecological and evolutionary factors. Previous studies showed that genetic diversity is associated with an individual's fitness (Fisher 1930; Hughes et al. 2008), allowing a species to persist and adapt in ever-changing environments (Lenormand 2002; Garant et al. 2007). Consequently, it is important to understand processes that influence genetic diversity in wild populations, while the maintenance of genetic diversity is a fundamental objective in wildlife conservation and management.

Genetic diversity can also have important ecological consequences within populations, and in turn, ecological factors may drive patterns of genetic diversity (Vellend and Geber 2005). The interaction between genetic diversity and ecological factors has been assessed in a few population-level studies in plants and animals (reviewed in Hughes et al. 2008). These studies showed important consequences of genetic diversity on fitness components, such as productivities in crop species (Crutsinger et al. 2006), susceptibility to environmental stresses and parasites (Tarpy 2003; Jones et al. 2004), or survival rate in animals (Rogell et al. 2010). However, relatively little is known about the causal relationships between ecological variables and genetic diversity (Reed and Frankham 2003). In addition to demographic processes, ecological and environmental factors can also play a role in shaping genetic diversity patterns (Gaggiotti et al. 2009), and these in turn may determine the likelihood of local adaptation and extinction in wild populations (Gilpin 1991; Hanski 1991, 1998). These issues are of importance in allowing a better understanding of microevolutionary processes as well as the development of appropriate conservation and management strategies (Reed and Frankham 2003).

To investigate how ecological factors are linked to local population size and genetic diversity, we used the house sparrow (*Passer domesticus*) as our study system. The house sparrow is one of the most numerous and widespread bird species in the world and is closely associated with human settlements (Anderson 2006), with their favorite habitats being farmlands and built-up areas. While their natural range covers Eurasia, the Middle East, and North Africa, repeated introductions by humans in the Americas and Australia as well as extension of agricultural areas have caused rapid population expansion and colonization in all continents except the Antarctic (del Hoyo et al. 2009). Despite this colonization success, massive population declines have occurred in their natural range in Europe and in introduced populations in North America in the late twentieth century (Hole et al. 2002). A reason for this population decline is the increasing intensification of agricultural land use, which reduces food availability for house sparrows (del Hoyo et al. 2009). At a local scale, changes in population demographics due to increased adult mortality rate have been shown to be responsible for the local extinction of house sparrow populations in northern Norway (Ringsby et al. 2006).

Previous studies demonstrated that patterns of genetic diversity in house sparrow populations varied at different geographical scales, which may be a consequence of population demography and ecological factors. Populations in the native ranges and natural habitats have higher genetic diversity compared with introduced populations (Schrey et al. 2011) or populations in secondary-colonized habitats (i.e., urban areas) (Vangestel et al. 2011). A possible reason for these differences might lie in the lower dispersal rates or distances when compared with native populations. In contrast, relatively similar levels of genetic diversity and genetic homogeneity were found among Finnish house sparrow populations, implying a considerable dispersal rate in a contiguous landscape (Kekkonen et al. 2010). Even finer-scale patterns of genetic diversity were found in house sparrow populations along the coast of Norway with lower genetic diversity in island populations than that in mainland populations. This is probably because of population bottlenecks that are more important to shape genetic composition of island populations than mainland populations (Jensen et al. 2013). In addition, pathogen-mediated balancing selection can maintain a high level of adaptive genetic diversity at MHC loci of house sparrow populations with low neutral genetic diversity (Borg et al. 2011). Although the house sparrow is a species that adapts well to human settlements and farms, only a few studies have examined the impacts of ecological factors on local population size and genetic diversity (Vangestel et al. 2011).

In this study, we analyzed the effect of environmental factors on population size and genetic diversity of house sparrows. We used observational data to estimate abundance of house sparrows at 36 locations in a confined valley in southern France. In 12 of 36 surveyed locations in this study, we captured 891 sparrows and genotyped these individuals at a panel of 10 autosomal microsatellite markers. Environmental and landscape characteristics of farms were also collected. Based on these data, we (i) compared population size and genetic diversity between locations; (ii) analyzed the population genetic structure; and (iii) assessed whether patterns of genetic diversity were correlated with environmental variables.

## Materials and Methods

### Study site

**Figure 1:**
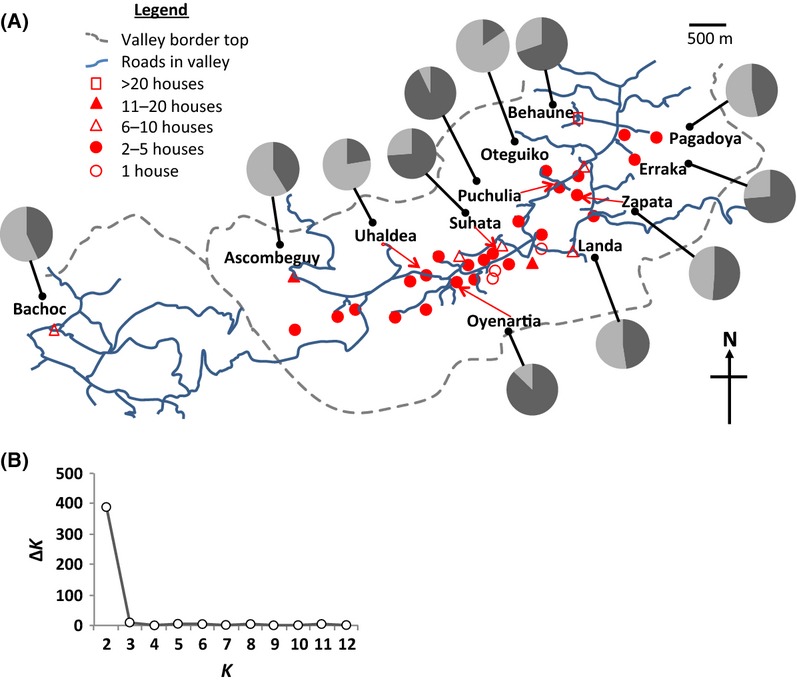
(A) Overview of the study site in Lantabat, southern France. The abundance of house sparrows (*Passer domesticus*) was counted at all 36 locations (expect Bachoc). For the genetic analyses, sparrows (*N* = 891 individuals) were caught at the 12 locations (names displayed on map). (B): Two genetic clusters were suggested based on the maximum value of the Delta *K* (Δ*K*) and the order rate of change in posterior likelihood Ln P (X/*K*) over 10 runs per *K*, using the software STRUCTURE. The proportion of population assignment of sparrows in relative to each of the two genetic clusters inferred by STRUCTURE is represented by black and gray cycles.

The data for this study were collected in a population of house sparrows in Lantabat (43°15′N, 1°07′W), about 40 km to the east of Biarritz, southern France, between May 2007 and March 2009. Lantabat is located in a confined, narrow valley that is surrounded by a continuous mountain ridge on three sides. The settlements in the valley are more or less evenly distributed along the valley's length and range in size from single houses (about 50) to three larger hamlets with up to 30 houses (Griesser et al. 2011). At most of these locations, sparrows are present year-round. In addition, we also collected data in one location outside the valley, 3 km to the west over the highest part of the ridge around the valley (Fig. 1). Traditional sheep herding on small meadows as well as cattle production dominates the agriculture in the valley. The only cereal crop cultivated in the valley is maize, which is carried out on a small scale and used as food for livestock. The maize cobs are stored in open outdoor storage frames, allowing the sparrows to feed on them.

### Assessment of population size

We sampled the number of sparrows using point counts in 36 locations between November 2007 and March 2008. For our surveys, we selected locations that were at least 100 m apart from each other (mean distance between locations = 252 m, min = 110 m, max = 850 m). The size of the surveyed locations varied between one and 30 buildings (mean = 4.6). We visited all these locations 10 times and counted the number of sparrows seen during 15-min intervals. We used a scan-sampling protocol where we scanned the location for sparrows once per minute. At each location, we selected the spot that gave the best view over the location, allowing us to assess the maximum number of sparrows. In the three larger locations with more than five buildings, three observers scanned simultaneously with a nonoverlapping observation range. While our sampling protocol did not allow for the counting of the maximum number of individual residents in a location, it gave a rough proxy for the number of sparrows in a location. In particular at locations with many individuals, this method will underestimate the number of sparrows, whereas it gives good abundance estimates for locations with no sparrows or only a few individuals present (Griesser et al. 2011).

### Effect of environmental variables on population size

We surveyed all locations in the study site and assessed whether the locations contained at least one active farm, a partially active farm (farmers which only had a few chickens and/or ducks on their farm, but no other livestock), or whether there was no active farm present. In addition, we assessed the numbers of livestock and food availability, which was measured as the degree of animal food spillage and serves sparrows as a main source of food (Hole et al. 2002). We categorized locations on a ordinal scale as locations without food spillage (i.e., locations without farms and thus no spillage of maize, chicken food, grains, manure, hay on the ground), locations with minor food spillage (locations with few animals that are fed, for example, chickens, ducks, but no livestock), locations with intermediate food spillage (modern farms with livestock some food spillage in a few places), and traditional farms with livestock with a large degree of food spillage across the whole location. We also counted the number of cats present in each location as they can prey upon sparrows. Linear models in SAS 9.3 (SAS Institute, Cary, NC) were used to investigate the effect of environmental variables on the mean and maximum number of sparrows observed in the 36 locations. We present minimum adequate models, where we used a backward stepwise regression procedure to remove nonsignificant factors from the initial full model (Crawley 2002).

### Blood sample collection and laboratory procedures

To assess house sparrow genetic diversity in these locations, we selected 12 locations with varying number of sparrows present, which were at least 200 m apart from each other (Fig. 1A). In each of these locations, we captured sparrows using mist nets across several sessions between October and February in years 2007–2009 (details given in Table 1). All captured birds were marked using an individually numbered metal band. An approximate volume of 30 μL blood was taken from the brachial vein of each individual and preserved in 95% ethanol. Whole genomic DNA was extracted using a high-salt purification protocol (Paxton et al. 1996). DNA samples were stored at −20°C for further microsatellite genotyping. Capture, ringing, and sampling of blood from house sparrows were carried out under the license from CRBPO (Paris, France) and Direction Régionale de l'Environnement Aquitaine (license nr. 14/2009).

**Table 1:** Catching locations used in the study of genetic diversity of rural house sparrow populations monitored between 2007 and 2009 (see Figure 1 for geographic distribution of locations). Bachoc is located outside the valley of Lantabat and had thus the biggest distance to the nearest location

Location	No. of seasons	Catching days	Total no. of birds caught	No. of recaptures	Distance to nearest catching location (m)
Bachoc	2	15	239	85	3310
Ascombeguy	3	17	210	76	1700
Uhaldea	2	3	41	4	440
Oyenartia	1	2	29	0	440
Suhata	2	3	42	3	905
Landa	1	2	38	5	910
Zapata	2	3	63	19	230
Oteguiko	2	3	66	14	310
Puchulia	1	1	27	0	230
Erraka	1	2	47	3	420
Behaune	2	3	196	11	910
Pagadoya	2	3	95	3	420

Twelve autosomal microsatellite loci were divided into two multiplex sets: Pdoμ1, Pdoμ3, Pdoμ5, Pdoμ9, and Ase18 (Neumann and Wetton 1996; Griffith et al. 1999b) as set one; and Pdo10, Pdo16. Pdo17, Pdo19, Pdo22, Pdo27, and Pdo40 (Dawson et al. 2012) as set two. Each set was amplified independently using Qiagen Multiplex mix (Qiagen, Hilden, Germany). PCR amplification was carried out in a 10-μL reaction volume using a multiplex protocol with 5 μL Multiplex PCR Master Mix (QIAGEN), 1 μL primer mix, 3 μL RNASE-free water, and 1 μL DNA sample. The PCRs were performed on a thermal cycler (Unocycler 2007 VWR®, Radnor, PA or Applied Biosystem®, Carlsbad, CA GeneAmp 2700) using the following thermal program: one denaturation step at 95°C for 15 min followed by 35 cycles at 94°C for 30 sec, 57°C for 90 sec, 72°C for 30 sec, and a final extension at 72°C for 10 min. Fragment analysis was carried out using MegaBACE 1000 DNA analyzer (Amersham® life science, Uppsala, Sweden). MegaBACE ET550-R size standard was used for the multiplex set1, and ET400-R size standard was used for multiplex set 2. Fragments of each individual were scored using MegaBACE Genetic Profiler software (Amersham Bioscience® V2.2, Uppsala, Sweden). A total of 891 house sparrows were genotyped. The loci Pdoμ9 and Pdo17 showed low amplification success with more than 20% missing genotypes and thus were removed from the further analysis (Table 2).

**Table 2:** Results of general linear models testing the effect of environmental variables on (A) maximum number of sparrows recorded in each location (*R*^2^ of model = 0.55) and (B) mean number of sparrows recorded in each location (*R*^2^ of model = 0.77)

Source	df	Type III SS	Mean square	*F* value	*P*-value
(A)					
Intercept	1	854.34	854.34	11.93	0.001
Food abundance	3	1314.72	438.24	6.12	0.002
Location inhabited	2	466.81	233.4	3.26	0.05
(B)					
Intercept	1	119.64	119.64	11.65	0.0021
Food abundance	3	291.11	97.03	9.45	0.0002
Size of location	11	350.9	31.9	3.11	0.008
Active farm in location	2	90.47	45.23	4.41	0.02

### Estimation of genetic diversity

Due to the fact that house sparrows maintain large population sizes, the presence of null alleles was reported in previous studies (Neumann and Wetton 1996; Griffith et al. 2007). We tested the allelic dropout and false alleles using Micro Checker, version 2.2.3 (Van Oosterhout et al. 2004), and estimated null allele frequencies for each locus in each location using the EM algorithm with the program FreeNA (Chapuis and Estoup 2007). We tested deviations from Hardy–Weinberg equilibrium (HWE), genotypic equilibrium, and the inbreeding index *F*_IS_ across loci for each population and assessed its significance based on 10,000 permutations in each location with Arlequin, version 3.5 (Excoffier and Lischer 2010). The same program was used to calculate the number of alleles (*N*_A_) and observed (*H*_O_) and expected heterozygosities (*H*_E_) in each population location. In addition, allelic richness (*A*_R_) was estimated using FSTAT, version 2.9.3.2 (Goudet 2002). We calculated multilocus population-specific *F*_ST_ values (Balding and Nichols 1995), which is an index to measure the level of genetic differentiation between a local population and within the entire population, using GESTE, version 2.0 (Foll and Gaggiotti 2006). Significance levels were adjusted for multiple testing using the sequential Bonferroni procedure (Rice 1989).

### Population genetic structure

We applied four different approaches to explore population substructure among the 12 locations. First, we estimated population substructure using principal component analyses (PCAs) based on microsatellite genotypes using with GenoDive, version 2.0b23 (Meirmans and Van Tienderen 2004) and visualized the results in Excel. This exploratory method allows multilocus genetic differentiation among individuals to be visualized. Secondly, we compared genetic differentiation between locations by calculating pairwise *F*_ST_ using the Weir and Cockerham estimator (Weir and Cockerham 1984) in Arlequin. Significance was obtained based on 10,000 permutations, with significance levels adjusted for multiple testing using the sequential Bonferroni corrections. An alternative estimator, Jost's *D*_est_ (Jost 2008), was also applied to calculated pairwise genetic differentiation because *F*-statistics may derive biased results when used for calculation of genetic differentiation using highly polymorphic microsatellite markers (Hedrick 2005; Jost 2008). Pairwise *D*_est_ values and associated significance levels were obtained on the basis of 10,000 permutations using GENALEX, version 6.5 (Peakall and Smouse 2012). Thirdly, we carried out spatial analysis of molecular variance implemented in SAMOVA, version 1.0 (SAMOVAs, Dupanloup et al. 2002) in order to define groups of populations that are maximally differentiated from each other (with maximum *F*_CT_ value) and genetic homogeneous between populations within a group (with minimum *F*_SC_ value). In SAMOVAs, all possible groupings were assessed, and statistical significance was tested by 1024 permutations. We further identified the number of genetic clusters (*K*) using the Bayesian admixture model with LOCIPRIOR option and correlated allele frequencies implemented in STRUCTURE, version 2.3.4 (Pritchard et al. 2000; Falush et al. 2003). We performed one million Markov chain Monte Carlo (MCMC) repetitions and a burn-in of 200,000 repetitions with ten independent runs each for *K* = 1–13. The most likely number of genetic clusters was determined on the basis of the ad hoc statistics described in Evanno et al. (2005) using STRUCTURE Harvester, version 0.6.8 (Earl 2011).

### Effect of geographic distance and environmental factors on genetic structure

To test for an association of genetic differentiation and geographical distances, that is, isolation by distance (IBD), we regressed linearized genetic differentiation between locations, measured as *F*_ST_/(1−*F*_ST_) (Rousset 1997), and geographical distances using Mantel tests implemented in GENALEX. Geographical distances were measured as the logarithm of geographical distance in meters between pairs of locations. The significance of the association was based on 9999 permutations using GENALEX.

Secondly, we tested for spatial genetic structure in house sparrows at a fine scale using spatial autocorrelation analyses with several distance class sizes based on microsatellite genotypes (Smouse and Peakall 1999). This method allows the global autocorrelation coefficient (*r*) among pairs of individuals at overlapping distance classes to be calculated. The autocorrelation coefficient, varying between −1 and 1, is a measure of pairwise genetic similarity between any pair of individuals within each distance class, relative to the overall genetic similarity. We calculated r among 891 samples at ten overlapping distance classes. The first distance class size was 0–500 m and increased by 500 m until 0–6000 m. We tested for the significance of observed *r*-vales by comparing it with a null distribution (*r* = 0). The 95% confidence intervals of each distance class of this null distribution were obtained using 999 permutation among individual genotypes within the given distance class. One thousand bootstrapping procedures over 10 loci were used to generate 95% standard errors around observed *r*-values.

To analyze the effects that different environmental factors may have on the genetic structure of house sparrows, we used a hierarchical Bayesian approach implemented in GESTE. This approach computes population-specific *F*_ST_ values and relates these values to specified environmental factors using a generalized linear model. The independence of environmental factors was tested using Spearman's rank correlation tests, and four factors were removed (food sources, cluster size, locations with sparrows in between and distance to next ringing location) showing significant correlations. The remaining five factors (livestock diversity, occurrence of cats, food abundance, distance to nearest woodland, and distance to nearest location) led to alternative 32 models (2^5^ optional models) that were considered in the simulations. The probabilities of each model were generated using a reversible jump MCMC approach by estimating the number of times that a linearized algorithm of the relationship between population-specific *F*_ST_ and environmental factors was visited by each model. GESTE eventually detects the model with the highest posterior probability that best explains the genetic structure. We performed 10 pilot runs of 1000 iterations to obtain the parameters of the proposal distributions used by the MCMC implemented in GESTE. We further applied an additional burn-in of 50000 iterations and a thinning interval of 20. All estimates were derived from a sample size of 10000. Each analysis was executed for three independent replicates to ensure consistency of results.

## Results

### Sparrow abundance

The mean and maximum number of sparrows varied across the locations; in some locations, we never observed sparrows (*N* = 14 locations), and in others, up to 51 individuals were observed. Both the mean and maximum number of sparrows observed in a location depended on farming practices and food availability (Table 1). Sparrows were more abundant in locations with active farms and a high degree of food spillage, which had the strongest effect on sparrow numbers explaining 46% of variation in the mean number of observed individuals and 47% of the variation in the maximum number of observed individuals (Table 2).

### Genetic diversity

Genetic diversity varied across the 12 locations (mean observed heterozygosity: 0.73–0.80) and was lower than expected (mean expected heterozygosity 0.85–0.90). Significant heterozygote deficits were observed in 51 of 187 locus-specific tests. Twenty-four of 540 tests (45 pairwise comparisons × 12 locations; 4.4%) showed significant deviations from linkage disequilibrium after Bonferroni corrections, but no systematic pattern occurred either between specific pairs of loci or population. In all 12 locations, the inbreeding coefficient *F*_IS_ was significantly higher than expected and ranged from 0.09 to 0.16. We found no evidence of genotyping error of stuttering and large-allele dropout, but the presence of null alleles at all loci was suggested by Micro Checker. The null allele frequencies were low in most loci (0.78–6.64%), but locus Pdo10 and Pdo22 exhibited a high level of null alleles (14.33% and 18.31%, respectively). Excluding these two loci slightly decreased the *F*_IS_ values (0.08–0.15), but all these values were still significantly larger than zero (data not shown). Thus, it seems these two loci were not the only cause of heterozygosity deficits, and therefore, all 10 loci were retained to calculate genetic diversity indices and estimated genetic structure (Table 3). The overall loci were highly polymorphic ranging from 11 to 39 alleles per locus, and the average allelic richness ranged from 10.42 (in Puchulia) to 13.31 (in Pagadoya) among all 12 locations. Additionally, population-specific *F*_ST_ values ranged between 0.006 (Bachoc) and 0.05 (Puchulia).

**Table 3:** Genetic diversity estimates for house sparrow (*Passer domesticus*) from the 12 locations in Lantabat, southern France, based at 10 microsatellite loci. Indices shown are number of individuals (*N*), average number of alleles (*N*_A_), allelic richness (*A*_R_), observed (*H*_O_) and expected (*H*_E_) heterozygosities, and multilocus inbreeding coefficients (*F*_IS_). Values shown in bold indicate significant deviations from Hardy–Weinberg equilibrium after Bonferroni corrections

Locality	*N*	*N*_A_	*A*_R_	*H*_O_	*H*_E_	*F*_IS_	Population-specific *F*_ST_
Bachoc	183	23.50	13.01	0.78	0.89	**0.12**	0.0058 (0.0039–0.0078)
Ascombeguy	185	21.70	12.35	0.79	0.89	**0.11**	0.0136 (0.0104–0.0170)
Uhaldea	40	14.90	11.53	0.79	0.89	**0.09**	0.0217 (0.0143–0.0300)
Oyenartia	24	13.30	11.69	0.75	0.87	**0.15**	0.0257 (0.0154–0.0360)
Suhata	37	16.30	12.42	0.73	0.87	**0.15**	0.0210 (0.0138–0.0283)
Landa	31	13.90	11.74	0.76	0.89	**0.13**	0.0182 (0.0110–0.0267)
Zapata	61	19.10	13.12	0.80	0.90	**0.11**	0.0078 (0.0046–0.0114)
Oteguiko	54	17.50	12.31	0.76	0.88	**0.14**	0.0197 (0.0140–0.0258)
Puchulia	26	11.90	10.42	0.76	0.85	**0.11**	0.0481 (0.0327–0.0648)
Erraka	41	14.20	11.21	0.73	0.88	**0.16**	0.0321 (0.0225–0.0421)
Behaune	127	20.90	12.24	0.78	0.88	**0.13**	0.0174 (0.0133–0.0215)
Pagadoya	82	21.10	13.31	0.77	0.89	**0.13**	0.0081 (0.0054–0.0112)

### Population genetic structure

**Figure 2:**
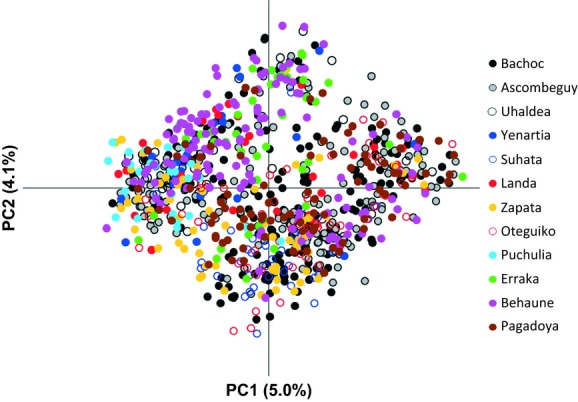
Plot of the first two component axes (PC1 and PC2) and the variance explained based on microsatellite genotypes of house sparrows (*Passer domesticus*) from the 12 locations in Lantabat, southern France.

The exploratory PCA method based on individual microsatellite genotypes revealed no distinct geographical substructure among individuals and large overlap between individuals from the different locations by plotting of the first two axes (Fig. 2). Overall, we found a low but significant genetic differentiation between different locations (global *F*_ST_ value: 0.017; *P* < 0.001). For pairwise genetic comparisons among locations, 60 of 66 comparisons among locations exhibited low but significant genetic differentiation (*F*_ST_ values ranged 0.004 from 0.06) (Table 4). A similar pattern was detected using Jost's *D*_est_, in which 58 of 66 comparisons had significant *D*_est_ values (ranged 0.04 from 0.37, Table S1). Additionally, pairwise *F*_*ST*_ and *D*_est_ values were significantly correlated (Pearson's correlation coefficient *r* = 0.98, *P* < 0.001).

**Table 4:** Pairwise geographic distances (above the line, in m) and pairwise genetic differentiation (below the line, *F*_ST_) among house sparrows (*Passer domesticus*) from the twelve locations in Lantabat, southern France. Values highlighted in bold represent significant genetic differentiation after Bonferroni correction

	Bachoc	A-beguy	Uhaldea	Oyenartia	Suhata	Landa	Zapata	Oteguiko	Puchulia	Erraka	Behaune	Pagadoya
Bachoc	–	3422.8	5175.9	5597.9	6428.6	7403.9	7682.5	7419.4	7497.8	8666.0	8236.5	9039.1
A-beguy	**0.006**	–	1784.2	2222.0	3009.4	3990.9	4270.1	4047.3	4096.7	5270.2	4931.7	5661.8
Uhaldea	**0.016**	**0.015**	–	442.9	1282.4	2232.6	2651.3	2579.6	2531.7	3685.1	3566.0	4115.8
Oyenartia	**0.025**	**0.025**	0.008	–	937.8	1832.4	2339.6	2349.7	2253.0	3373.9	3354.4	3817.8
Suhata	**0.018**	**0.025**	**0.040**	**0.046**	–	989.7	1402.9	1474.5	1332.5	2436.4	2479.2	2882.1
Landa	**0.011**	**0.017**	0.013	**0.019**	**0.035**	–	903.5	1352.6	1039.6	1762.6	2175.6	2225.8
Zapata	**0.009**	**0.012**	**0.015**	**0.022**	**0.011**	**0.017**	–	581.6	265.1	1035.5	1272.2	1479.4
Oteguiko	**0.010**	**0.017**	**0.028**	**0.036**	**0.016**	**0.017**	**0.012**	–	335.0	1268.1	1007.3	1619.8
Puchulia	**0.043**	**0.040**	0.012	0.013	**0.061**	**0.035**	**0.023**	**0.057**	–	1174.2	1191.6	1586.7
Erraka	**0.020**	**0.022**	**0.023**	**0.032**	**0.057**	**0.017**	**0.029**	**0.036**	**0.055**	–	980.3	463.6
Behaune	**0.016**	**0.019**	**0.014**	0.010	**0.045**	0.007	**0.026**	**0.027**	**0.038**	**0.016**	–	1021.5
Pagadoya	**0.004**	**0.011**	**0.018**	**0.031**	**0.026**	**0.012**	**0.014**	**0.011**	**0.046**	**0.026**	**0.020**	–

The SAMOVA results showed that *F*_CT_ values kept increasing with rising numbers of genetic clusters, and reached the maximum value when 11 separate groups were assumed (Figure S1). In this case, however, the only locations that yielded a significant *F*_SC_ value were Bachoc and Ascombeguy, including more than one group member. These results indicate that there was no meaningful grouping suggested by SAMOVA, and each location represents a distinct subpopulation.

The Bayesian clustering approach implemented in STRUCTURE suggested *K* = 2 as the most likely genetic cluster based on the Evanno's method (Fig. 1B). Evidence of admixture found that each location contains individuals from the two genetic clusters with different proportions (Fig. 1A).

### Effect of geographic distance and environmental factors on genetic structure

**Figure 3:**
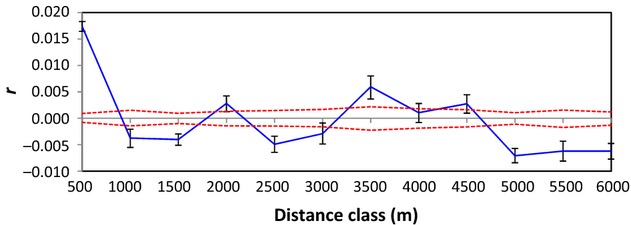
Correlograms of spatial autocorrelation plots based on 10 loci of 891 house sparrows (*Passer domesticus*) from the 12 locations in Lantabat, southern France. Autocorrelation values (*r*) are represented by the solid line. The red dashed line represents the 95% confidence limits around *r* of zero determined by 999 *r* permutations of the data. Error bars represent the bootstrap 95% confidence limits around the estimates of *r* for each distance class.

Based on the findings from the population genetic analyses, we tested how geographic distance and ecological factors affected the genetic differentiation between locations. The results of the Mantel tests did not support a significant correlation between populations and geographical distance (*R*^2^ = 0.035, *P* = 0.12). However, analyses of spatial autocorrelation indicated local genetic substructuring at a very fine scale (Fig. 3). We found that the autocorrelation coefficients (*r*) between individuals were significantly positive in the first distance class (500–1000 m) and the signals of genetic similarity diminished after this distance interval and fluctuated randomly.

The relative importance of environmental factors on the genetic variability between locations was assessed using the approach implemented in the software GESTE. Food availability appears to be the most important factor in explaining genetic variation between locations because it had the highest cumulative posterior probability (Table 5A). The model with food availability explained 30% of the genetic variation found between locations and had a posterior probability of about 0.3 (Table 5B). The second highest posterior probability was assigned to the distance to the nearest sampling location (i.e., geographic distance between sampling locations). The model with this factor and food abundance received a posterior probability of about 0.2 (Table 5B). The remaining three factors (livestock diversity, occurrence of cats, and distance to woodland) had much lower scores, and these resulted in models with negligible values of posterior probabilities (<0.05).

**Table 5:** Analysis of genetic and environmental differentiation among house sparrows (*Passer domesticus*) from the twelve locations in Lantabat, southern France, using GESTE. (A) Sum of posterior probabilities of models that included given environmental factors indicating food availability with highest score; (B) posterior probabilities of the five most likely models overall 32 alternative models

(A) Factor	Sum of posterior probabilities
Food availability	0.607
Distance to nearest location	0.322
Livestock diversity	0.105
Occurrence of cats	0.100
Distance to woodland	0.069

(B) Model (Factor included)	Posterior probabilities
5 (food availability)	0.296
21 (distance to nearest location + food availability)	0.193
1 (constant)	0.189
2 (livestock diversity)	0.046
3 (occurrence of cats)	0.037

## Discussion

Understanding factors that determine population size and genetic diversity are of crucial importance for ecology in general and conservation genetics in particular. Our results show that ecological and landscape features affect both the abundance and the genetic diversity of house sparrows in locations in our study site. Locations with higher food abundance harbor larger sparrow populations, independently of the geographic distance to other locations (Table 2). However, we found the genetic variation at the different locations was dependent both on food abundance and geographic distance to the nearest location, which was inhabited by sparrows.

### Food abundance and population size

In many studies, food abundance has been demonstrated to influence the distribution and size of wild populations (Newton 1998; Benton et al. 2003). Accordingly, sparrows were more abundant at locations with a high level of food spillage, and an earlier study in this population found that food abundance directly influenced group size distributions (Griesser et al. 2011). The recent decline in house sparrow density in farmland habitats of Europe has also been linked to reduced food availability due to more cost-effective methods of handling agricultural crops and livestock feeding (Robinson et al. 2005). Our results show that reduced food availability leads to lower genetic diversity, which further might reduce the viability of small populations. The method used to estimate population sizes underestimates, in particular in large locations with many sparrows, the actual number of sparrows (e.g., 210 sparrows caught in Ascombeguy, but only a maximum of 40 counted). In contrast, the mismatch between the number of birds caught and observed was much smaller in locations with fewer sparrows (e.g., Uhaldea: 29 caught vs. 19 maximum sparrows observed). Thus, it is likely that our analyses actually underestimated the link between food abundance and genetic diversity.

### Genetic diversity in house sparrows

Based on multilocus microsatellites, we estimated genetic diversity within house sparrows in 12 locations at a microgeographical scale in southern France. The observed magnitude of genetic diversity measures was comparable with previous studies at larger geographical scales (Kekkonen et al. 2011, Schrey et al. 2011), but higher than the island–coastal populations along the coast in Norway (Jensen et al. 2013), which is not surprising given the large effective population size of this species in its native range. We found positive values of the inbreeding coefficient (*F*_IS_), which deviated significantly from zero. The deficits in observed heterozygosities were retained, even after we excluded the two loci with high null allele frequency (Table 3). These results could be explained by the presence of genetic substructure leading to Wahlund effects (Wahlund 1928) or nonrandom mating due to inbreeding (Charlesworth and Charlesworth 1987) rather than the presence of null alleles. We suggest that genetic admixture rather than inbreeding may explain the observed patterns of two genetic clusters for two reasons. First, a Bayesian clustering method in STRUCTURE revealed evidence of admixture where individuals in each location descended from two ancestral groups. Secondly, in contrast to the genetic signature of inbreeding, which would be reflected in a reduced level of genetic diversity, the overall level of allelic richness and genetic diversity indices were high throughout all locations and not significantly different between the two inferred genetic groups (data not shown).

### Fine-scale genetic variation and environmental factors

Despite the fact that the house sparrow is on its way to becoming a behavioral and ecological model species, only a few studies have investigated genetic structure at a comparably small scale (Liker et al. 2009). The magnitude of genetic differentiation assessed in this study is smaller than the average differentiation between countries within the native range of the house sparrow (Schrey et al. 2011). At a large geographical scale, population structure was probably built-up by distinct evolutionary history and maintained by limited dispersal at a continental scale. In contrast, Kekkonen et al. (2010) found no evidence of population substructure within Finnish house sparrows. Evidence of panmixia is not rare in bird species and is often interpreted as a consequence of frequent population admixture (Kekkonen et al. 2010; Liu et al. 2012). Unlike these studies that used a relatedness estimator, we applied *F*-statistic coupled with Bayesian analyses to unravel potential population structure and the underlying environmental drivers. We found low and significant genetic differentiation in house sparrows at the 12 locations and evidence for the presence of two genetic clusters. However, these genetic clusters did not correspond with the geographical locations of farms, and populations at each location were mixing with individuals from two different genetic ancestors. Moreover, the genetic variation is correlated with food availability and may be partially due to geographical distance to the nearest location. Taken together, our study shows that house sparrows populations can be genetically differentiated at a surprisingly fine geographical scale.

Although we did not find evidence of isolation-by-distance between locations, we found a positive autocorrelation at very short distances (500–1000 m), but this signal is missing over larger distances (1–6 km; Fig. 3). This result strengthens the idea that the observed pattern of genetic diversity at the study site partially depended on geographical distance. Despite being able to move large distances, house sparrows are generally very sedentary after the juvenile dispersal phase (Skjelseth et al. 2007; Pärn et al. 2012), which is confirmed by ring recoveries from our study site. While movements between nearby locations occur frequently (e.g., from Zapata to the nearby Puchulia, a location with a high food abundance), which is also reflected in the results from GESTE (Table 5), we only recorded seven between-location movements with an average distance of 855 m (range 261–2479 m). When excluding movements between Zapata and Puchulia, we only recorded four between-location movements, despite the fact that we recaptured 193 individuals (2.1% of all recaptured birds, 0.4% of all caught birds) and that a substantial proportion of birds caught were juveniles (at least 20%, M. Griesser and Y. Liu unpubl. data). Thus, our data suggest that once birds had settled after juvenile dispersal, they were highly faithful to their location even outside the breeding season in spite of ample dispersal opportunities.

The most interesting finding of this study is that food availability was found to influence the observed pattern of genetic variation found between locations, as well as population size. Although fine-scale population structure driven by ecology and habitat structure has been reported in birds (Edelaar et al. 2012; Porlier et al. 2012), the influence of food availability on genetic variability has to our knowledge not so far been reported. Given that food abundance is of importance for the survival in birds in an agricultural landscape (Benton et al. 2003), the observed link between genetic variability and food abundance might reflect preferential settlement of aggregated groups in food-rich location and/or improved survival prospects. We suspect that lower food availability might limit flock size per se and *vice versa*. This in turn shapes genetic composition and thus intrapopulation genetic variation (Jensen et al. 2013).

### Conservation implications

Although the house sparrow is categorized as a species of least concern according to the IUCN Red List (BirdLife International 2013), sparrow populations have experienced dramatic declines since 1980, in both urban and rural areas of its native range in Europe (Anderson 2006). Our findings provide several conservation implications for this species. Firstly, the house sparrow is one of the most broadly distributed birds across the world and is a common resident in both agricultural and urban areas (del Hoyo et al. 2009) and thus a key indicator species of the health of these ecosystems. Given that house sparrows were very successful in colonizing new habitats and exhibit extensive phenotypic diversity within both the native and introduced ranges (Anderson 2006), this species is an important model species in ecological and behavioral studies (Griffith et al. 1999a; Tóth et al. 2009; Kekkonen et al. 2010). There is also relatively little known on fine-scale population processes and potential consequences of species that have colonized human settlements (Vangestel et al. 2011). Due to the fact that human-induced environmental changes constantly influence microevolutionary processes (Garant et al. 2004), it is important to understand the influence of local environmental factors on the variation of population size and genetic diversity. This can extend our knowledge of local adaption and population persistence, allowing decision makers to carry out scientifically informed conservation efforts.

Secondly, this study successfully links ecological factors to species abundance and genetic diversity. This implies that local environmental variables may substantially influence the population viability of sedentary species. Our results suggest that food abundance had the predominant influence on genetic diversity, while increasing distance to the next sparrow sampling location reduced genetic diversity. These findings are in line with studies in rural England which found that reduced food availability increased mortality, which in turn restricted dispersal between populations (Hole et al. 2002). Agricultural intensification during the last 60 years led to a replacement of winter stubble with autumn sowing (Robinson et al. 2005). Therefore, effective landscape-level conservation efforts should consider measurements that increase food availability outside the breeding season, which can effectively regulate population size (Arcese and Smith 1988).

Finally, our study provides an example of how population size and neutral genetic diversity vary across a contiguous agricultural landscape. As population size alone cannot always be a proxy to assess the viability of subpopulations (Frankham et al. 2002), combined conservation genetic approaches that illustrate genetic diversity can help to make firm evaluations on the risk of local extinction. If the observed low genetic diversity of subpopulations were due to their actual low effective population size, they would be more likely to be influenced by demographic and environmental stochasticity and thus prone to genetic drift (Ellstrand and Elam 1993). For organisms that do not disperse widely (such as house sparrows) (Pärn et al. 2012) and for which immigration may not counteract the effect of genetic drift in small populations, conservation management should focus on improving ecological conditions for small subpopulations. Our results show that both physical (distance) and biotic (food) factors influence genetic diversity in house sparrows, and in particular, the later of these factors provides a straight forward conservation tool to manage small populations. This finding highlights the importance to combine ecological and genetic data to understand microevolutionary processes.

## Data Accessibility

Microsatellite genotypes: Dryad doi:10.5061/dryad.n8379.

## Supporting Information

**Figure S1.** Plots of indexes of genetic differentiation among house sparrows (*Passer domesticus*) from the 12 locations in Lantabat, southern France, using the spatial analysis of molecular variance (SAMOVA) framework.

**Table S1.** Pairwise genetic differentiation using the estimator of Jost's *D*_est_ among house sparrows (*Passer domesticus*) from the 12 locations in Lantabat, southern France (below the diagonal line), and significant values (above the diagonal line). Values highlighted in bold represent significant genetic differentiation after Bonferroni correction.
