# Supplementary material for: Environmental factors influence both abundance and genetic diversity in a widespread bird species
Source: Ecol Evol. 2013 Oct 28;3(14):4683–95. doi: 10.1002/ece3.856 (PMC3867904; doi:10.1002/ece3.856)
Supplement: Supplementary file 1 [file ece30003-4683-SD1.docx]

**Supporting information**

**Environmental factors influence both abundance and genetic diversity in a widespread bird species**

Yang Liu ^1,2^*, Simone Webber ^3^, Katharine Bowgen ^4^, Lucie Schmaltz ^5^, Katharine Bradley ^6^, Peter Halvarsson ^7^, Mohanad Ali ^7,8^ , Michael Griesser ^9,10^

1 State Key Laboratory of Biocontrol and School of Life Sciences, Sun Yat-sen University, Guangzhou 510275, China

2 Computational and Molecular Population Genetics, Institute of Evolution and Ecology, University Bern, Balzerstrasse 6, CH-3012 Bern, Switzerland

3 Centre for Ornithology, University of Birmingham, Edgbaston, Birmingham BT15 2TT, UK

4 Centre for Conservation Ecology and Environmental Change, School of Applied Sciences, Bournemouth University, Talbot Campus, Poole, Dorset, BH12 5BB, UK

5 Animal Ecology Group, Centre for Ecological and Evolutionary Studies, University of Groningen, P.O. Box 11103, 9700 CC Groningen, The Netherlands.

6 10 Springhill Close, London, SE5 8AJ, UK

7 Section of Animal Ecology, Department of Ecology and Evolution, Evolutionary Biology Centre, Uppsala University, SE-75236 Uppsala, Sweden

8 Department of Biology, College of Science, University of Hail, PO 2440, Hail, Saudi Arabia

9 Department of Ecology, Swedish University of Agricultural Sciences, Uppsala, Sweden

10 Anthropological Institute and Museum, University Zürich, 8057 Zürich, Switzerland

* Corresponding author: State Key Laboratory of Biocontrol and School of Life Sciences, Sun Yat-sen University, Guangzhou 510275, China

E-mail: [liuy353@mail.sysu.edu.cn](mailto:liuy353@mail.sysu.edu.cn)

Figure S1. Plots of indexes of genetic differentiation among house sparrows (*Passer domesticus*) from the 12 locations in Lantabat, Southern France using the Spatial Analysis of Molecular Variance (SAMOVA) framework. Genetic differentiation were partitioned into variation between groups (*F*_CT_), among populations within groups (*F*_SC_) and within populations (*F*_ST_) against numbers of groups (*K*=1-11). When there is one group, only the global *F*_ST_ was shown.

Table S1. Pairwise genetic differentiation using the estimator of Jost’s *D*_est_ among house sparrows (*Passer domesticus*) from the 12 locations in Lantabat, Southern France (below the diagonal line), and significant values (above the diagonal line. Values highlighted in bold represented significant genetic differentiation after Bonferroni correction.

|  |  |  |  |  |  |  |  |  |  |  |  |  |
| --- | --- | --- | --- | --- | --- | --- | --- | --- | --- | --- | --- | --- |
|  | Bachoc | A-beguy | Uhaldea | Oyenartia | Suhata | Landa | Zapata | Oteguiko | Puchulia | Erraka | Behaune | Pagadoya |
|  |  |  |  |  |  |  |  |  |  |  |  |  |
|  |  |  |  |  |  |  |  |  |  |  |  |  |
| Bachoc | --- | 0.0001 | 0.0001 | 0.0001 | 0.0001 | 0.0002 | 0.0001 | 0.0001 | 0.0001 | 0.0001 | 0.0001 | 0.0012 |
| A-beguy | **0.0445** | --- | 0.0001 | 0.0001 | 0.0001 | 0.0001 | 0.0001 | 0.0001 | 0.0001 | 0.0001 | 0.0001 | 0.0001 |
| Uhaldea | **0.1142** | **0.1138** | --- | 0.0107 | 0.0001 | 0.0040 | 0.0001 | 0.0001 | 0.0122 | 0.0001 | 0.0001 | 0.0001 |
| Oyenartia | **0.1874** | **0.1892** | 0.0674 | --- | 0.0001 | 0.0006 | 0.0001 | 0.0001 | 0.0047 | 0.0001 | 0.0011 | 0.0001 |
| Suhata | **0.1217** | **0.1688** | **0.2646** | **0.3090** | --- | 0.0001 | 0.0017 | 0.0001 | 0.0001 | 0.0001 | 0.0001 | 0.0001 |
| Landa | **0.0770** | **0.1163** | 0.0768 | **0.1193** | **0.2241** | --- | 0.0001 | 0.0001 | 0.0001 | 0.0001 | 0.0052 | 0.0005 |
| Zapata | **0.0683** | **0.0976** | **0.1002** | **0.1743** | 0.0625 | **0.1197** | --- | 0.0001 | 0.0001 | 0.0001 | 0.0001 | 0.0001 |
| Oteguiko | **0.0867** | **0.1305** | **0.2124** | **0.2573** | **0.1099** | **0.1184** | **0.1074** | --- | 0.0001 | 0.0001 | 0.0001 | 0.0001 |
| Puchulia | **0.2718** | **0.2622** | 0.0571 | 0.0798 | **0.3449** | **0.2006** | **0.1285** | **0.3694** | --- | 0.0001 | 0.0001 | 0.0001 |
| Erraka | **0.1400** | **0.1621** | **0.1510** | **0.2111** | **0.3620** | **0.1088** | **0.2000** | **0.2659** | **0.3106** | --- | 0.0001 | 0.0001 |
| Behaune | **0.1239** | **0.1462** | **0.1006** | 0.0732 | **0.3065** | 0.0507 | **0.1966** | **0.2042** | **0.2322** | **0.1085** | --- | 0.0001 |
| Pagadoya | 0.0257 | **0.0838** | **0.1457** | **0.2288** | **0.1885** | **0.0853** | **0.1142** | **0.0957** | **0.3095** | **0.1888** | **0.1520** | --- |
|  |  |  |  |  |  |  |  |  |  |  |  |  |
